# Potential geographical distribution and environmental explanations of rare and endangered plant species through combined modeling: A case study of Northwest Yunnan, China

**DOI:** 10.1002/ece3.7999

**Published:** 2021-09-04

**Authors:** Pengcheng Ye, Guangfu Zhang, Xiao Zhao, Hui Chen, Qin Si, Jianyong Wu

**Affiliations:** ^1^ Nanjing Institute of Environmental Sciences Ministry of Ecology and Environment of the People’s Republic of China Nanjing China; ^2^ Jiangsu Key Laboratory of Biodiversity and Biotechnology School of Life Sciences Nanjing Normal University Nanjing China

**Keywords:** biodiversity conservation, environmental explanations, geographically weighted regression, habitat suitability, MaxEnt, Northwest Yunnan, potential geographical distribution

## Abstract

In recent decades, due to the effect of climate change and the interference of human activities, the species habitat index has fallen by 2%. Studying on the geographical distribution pattern and predicting the potential geographical distribution of species are of great significance for developing scientific and effective biodiversity conservation strategies. Plenty of rare and endangered species that need immediate conservation are distributed in Northwest Yunnan. In this regard, this research is conducted in the purpose of predicting the potential geographical distribution of 25 rare and endangered plant species in Northwest Yunnan and analyzing the explanation capabilities of various environmental factors on the potential geographical distribution patterns of these species. Initially, the ecological niche model MaxEnt was employed to predict the potential geographical distribution of target species. Following that, the superposition method was applied to obtain the potential geographical distribution pattern of species richness on the spatial scale of the ecological niche model with a resolution of 0.05° × 0.05°. Ultimately, geographically weighted regression (GWR) model was adopted to investigate the explanation capabilities of various environmental parameters on the potential distribution patterns. The research results showed that the average value of the area under the receiver operating curve (AUC) of each species was between 0.80 and 1.00, which indicated that the simulation accuracy of the MaxEnt model for each species was good or excellent. On the whole, the potential distribution area for each species was relatively concentrated and mainly distributed in the central‐western, central‐eastern and northern regions of Northwest Yunnan. In addition, the potential distribution areas of these species were between 826.33 km^2^ and 44,963.53 km^2^. In addition, the annual precipitation (Bio12), precipitation of coldest quarter (Bio19), and population density (Pop) made a greater contribution to the species distribution model, and their contribution values were 25.92%, 15.86%, and 17.95%, respectively. Moreover, the goodness‐of‐fit *R* ^2^ and AIC value of the water model were 0.88 and 7,703.82, respectively, which indicated the water factor largely influenced the potential distribution of these species. These results would contribute to a more comprehensive understanding of the potential geographical distribution pattern and the distribution of suitable habitats of some rare and endangered plant species in Northwest Yunnan and would be helpful for implementing long‐term conservation and reintroduction for these species.

## Graphical abstract

- MaxEnt model was used to predict the potential distribution of 25 rare, endangered, and/or national protected species in Northwest Yunnan, China. We calculated that the potential distribution area of these species was between 826.33 km^2^ and 44,963.53 km^2^. The potential distribution areas of these species increased with the decline of habitat suitability.
- GWR model was employed to investigate the explanation capabilities of various environmental parameters on the potential distribution patterns of these target species. Water factor largely affected the potential distribution pattern of these target species.

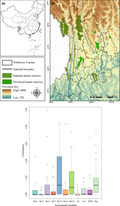

## 1 INTRODUCTION

The geographical distribution pattern of species and utilizing species distribution models (SDMs) to predict the potential geographical distribution of species are one of the hot research issues in the fields of biogeography and biodiversity conservation (Gaston, 2000; Ning et al., 2020; Tripathi et al., 2019a; Zhang & Ma, 2008). Studying on the geographical distribution pattern and predicting the potential geographical distribution of species are of great significance for developing effective biodiversity conservation strategies (Lazo‐Cancino et al., 2020; Zhang et al., 2019), preventing and managing the spread of invasive alien species diffusion (Fernandes et al., 2019), and assessing the impact of climate change on species distribution (Lazo‐Cancino et al., 2020), and they are also an effective means of protecting and managing some rare and endangered species.

It is stated by the Living Planet Report 2020 (https://www.wwf.org.uk/press‐release/living‐planet‐report‐2020) that, from 2000 to 2018, the species habitat index (i.e., a single metric that conveys change in suitable habitat available to all species over time) has fallen by 2%, which indicated that the available habitats of species have shown a strong and general downward trend. Northwest Yunnan of China is one part of the global biodiversity hot spots (Myers et al., 2000; Ye, Chen, et al., 2020; Ye, Zhang, et al., 2020). Plenty of rare, endangered, threatened, and endemic species that need immediate conservation are distributed in this area (Yang et al., 2017). In recent decades, due to the interference of human activities (e.g., mining and collecting herbs) and the influence of the external natural environment, especially climate change, the populations and distribution area of some rare, endangered, and threatened species have been decreasing (Yu et al., 2014). Therefore, Northwest Yunnan has become an ideal region to discuss the distribution pattern of species diversity and simulate the distribution of potential suitable habitats. In addition, some species have the characteristics of geographical isolation and narrow distribution (Wang et al., 2013); as a result, their survival and development are faced with severe threats. Therefore, it is urgent to protect these rare and endangered species and their suitable habitats in this area. In order to take reasonably and effectively protect actions, the geographical distribution of these species must be identified first. However, at present, it is still a tough issue to figure out the potential geographical distribution and main limited environmental factors for these rare and endangered species in Northwest Yunnan.

The maximum entropy (MaxEnt) model is a species distribution model based on the environmental factors matching method and is often used to predict the potential geographical distribution of species (Phillips et al., 2006; Phillips & Dudík, 2008; Zhang et al., 2019). The MaxEnt model employs the data of species distribution locations and environmental variables to jointly simulate the potential geographical distribution of species and has many advantages over other species distribution models, for example, easy operation and use, high simulation accuracy, and good performance with incomplete datasets (Li et al., 2020). Currently, the MaxEnt is the most widely used species distribution model (Gong et al., 2015; Merow et al., 2013; Ning et al., 2018). The research results of Hernandez et al. (2006) and Deb et al. (2017) both showed that in the case of few geographical locations (<10), even as low as 4 or 5, the MaxEnt model can still produce effective prediction results with high accuracy. At present, the model has been applied to simulate and predict the potential geographical distribution of endemic species (e.g., *Impatiens hainanensis*; Ning et al., 2020), national protected species (e.g., *Phellodendron amurense*; Huang et al., 2018), and many other key species.

It is helpful to improve the scientific understanding of the spatial relationship between species and environment by understanding the impact of different types of environmental factors on the potential geographical distribution of species. The spatial distribution pattern of species diversity is related to variations in environmental factors (e.g., latitude, elevation, and climate) (Tripathi et al., 2019a). However, the relationship between them often has spatial nonstationarity (i.e., relationship between independent and dependent variables will change with geographical location) (Gouveia et al., 2013). Geographically weighted regression (GWR) model, which is an extension of traditional regression model (e.g., ordinary least squares, OLS) (Ștefănescu et al., 2017; Tripathi et al., 2019a, 2019b; Xue et al., 2020), has become one of the crucial spatial heterogeneity modeling tools (Lu et al., 2020). In recent years, many domestic and foreign scholars have carried out in‐depth and extensive research in various fields by using GWR model, including social environmental factors and regional economy, regional house prices and pollution (McCord et al., 2018; Xu et al., 2019), the impacts of environmental heterogeneity and land‐use change on wild animal distribution (Liu et al., 2019; Wang et al., 2020; Xue et al., 2020), and vegetation activity and climate change (Gao et al., 2019). However, there are few studies that in combination MaxEnt with GWR models to analyze the potential geographical distribution and explore environmental explanations for some rare and endangered plant species, especially in biodiversity hot spot areas.

In this study, we analyzed the potential geographical distribution and environmental explanations of 25 rare and endangered plant species in Northwest Yunnan through combined modeling. Initially, the MaxEnt model was adopted to predict the potential geographical distribution of each species on the grid map with a resolution of 0.05° × 0.05° (Ye, Zhang, et al., 2020). Then, the potential geographical distribution of each species was overlaid which produced the potential geographical distribution pattern of species richness on the spatial scale of the ecological niche model with a resolution of 0.05° × 0.05°. Ultimately, GWR model was employed to investigate the explanation capabilities of various environmental factors on the potential geographical distribution patterns of these target species, and the main restrictive environmental factors were gained as a result. These research results would contribute to a more comprehensive understanding of the potential geographical distribution pattern of some rare and endangered plant species in Northwest Yunnan and would provide a scientific basis for the conservation and management of suitable habitat for many other key species in this region.

## 2 MATERIALS AND METHODS

### 2.1 Study area and species data

**FIGURE 1:**
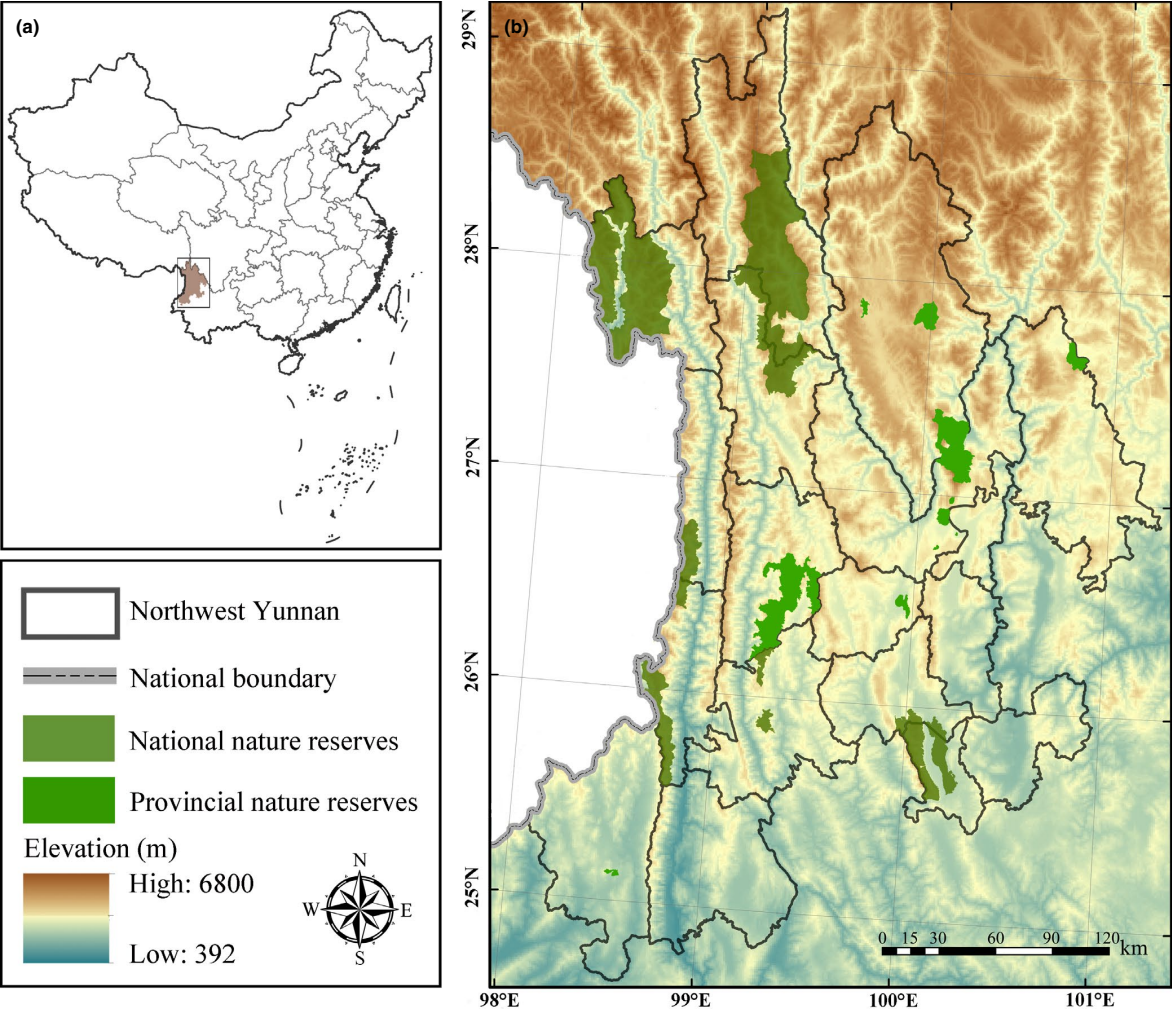
Map of the study area. (a) The location of Northwest Yunnan in China; (b) the topographic map of Northwest Yunnan and the distribution of national nature reserves (NNRs) and provincial nature reserves (PNRs) in this region

Northwest Yunnan is one part of the global biodiversity hot spots, which located in the mountains of southwest China, and lies to the northwestern part of Yunnan Province (Figure 1). The area is situated in the transition zone between the Qinghai–Tibetan Plateau and the Yunnan–Guizhou Plateau. As one of the three centers of origin and distribution of endemic species in China, the Northwest Yunnan region is located in the uplift and fold zone of the Himalayas in the Quaternary.

The geological history of this area is young, the movement is active, and the species differentiation is strong. A large number of endemic species have evolved and formed many endemic populations (He et al., 2020). In addition, the spatial variation of environmental factors makes the distribution pattern of species diversity more complicated. Massive variations in terrain and climate in Northwest Yunnan result in a rich and diverse special habitat environment, which provide habitats for special species, especially for rare and endangered species (Ye, Zhang, et al., 2020). In particular, alpine ecosystem and plateau lake ecosystem that provide habitats for a large number of rare and endangered species (Tao et al., 2016). The special terrain, diverse climate, and active geological history make this area become one of the most concentrated and abundant regions of biodiversity in China (Feng et al., 2010; Xue & Wu, 2016; Ye, Chen, et al., 2020).

In this study, we first integrated seven attributes and used them as criteria for comprehensively selecting rare and endangered plant species that need to be focused on and protected in Northwest Yunnan. After that, we selected 114 plant species, these species should (1) belong to rare and endangered plant species (refer to the IUCN threatened grade); (2) belong to the national protected plant species [refer to the National Key Protected Wild Plants List (the first), and the National Key Protected Wild Plants List (the second)]; and (3) at least have one of these attributes [refer to endemism, Plant Species with Extremely Small Populations (PSESP), The Convention on International Trade in Endangered Species of Wild Fauna and Flora (CITES), and The List of Key Protected Wild Plants in Yunnan Province (the first)] (Ye, Zhang, et al., 2020). Then, we collected georeferenced records for 114 species from two sources: the main digital herbarium in China and field survey (2010–2020) data for nearly a decade. By combining and accumulating data from these two sources, a total of 941 records of occurrence data were obtained. Finally, a total of 25 species (including 314 distribution records; Figure S1) from 114 key higher plant species (comprises 941 georeferenced records; Figure S1) were selected (see selection standards below). The selection was based on the combination of the following standards: (1) occurrence records: aiming at the continuous improvement of the MaxEnt model prediction accuracy, hence the number of species distribution records should not less than four (Deb et al., 2017); (2) simulation accuracy: MaxEnt model should have good or excellent simulation accuracy for included species (for more details, see section 2.4.1); and (3) spatial autocorrelation: There is no obvious spatial autocorrelation between occurrence data (Moran's *I* = 0.18, *p* > .05). The information used to construct the dataset (e.g., taxonomic level, threatened level, and georeferenced records) was obtained from field survey in nearly a decade and main virtual herbarium in China. For example, we get the taxonomic level and threatened level mainly from Flora of China (http://www.iplant.cn/frps) and Information System of Chinese Rare and Endangered Plants (ISCREP) (http://www.iplant.cn/rep/), respectively. In addition, we collected species distribution data through the Chinese Virtual Herbarium (http://www.cvh.ac.cn/) and Herbarium, Kunming Institute of Botany, CAS (http://www.kun.ac.cn/).

### 2.2 Environmental variables

Based on previous studies (Liu et al., 2019; Nieto et al., 2015; Ștefănescu et al., 2017; Zhang et al., 2019), 24 environmental variables were selected which may affect species distribution to model the current potential geographical distribution patterns (Table 1). These variables were divided into five groups according to their categories. After that, 24 environmental factors were resampled and reprojected to an equal‐area grid system with the same spatial resolution (0.05° × 0.05°) as species richness (Wang et al., 2018). Then, the ArcGIS 10.4 software (Esri; Redlands, California, USA) was employed to extract the raster data of environmental variables. In this study, the geographical coordinate system we used was WGS 1984.

**TABLE 1:** Environmental variables used to predict the potential geographical distribution of each species

Hypothesis	Environmental variable (Unit)	Abbreviation	Resolution	Time period	Data source
Energy availability	Annual mean temperature (℃)	Boi1	2.5 min	1970–2000	WorldClim version 2‐Bioclimatic variables (http://worldclim.com/)
	Mean diurnal range (℃)	Bio2			
	Isothermality (℃)	Bio3			
	Temperature seasonality (℃)	Bio4			
	Max temperature of warmest month (℃)	Bio5			
	Min temperature of coldest month (℃)	Bio6			
	Temperature annual range (℃)	Bio7			
	Mean temperature of wettest quarter (℃)	Bio8			
	Mean temperature of driest quarter (℃)	Bio9			
	Mean temperature of warmest quarter (℃)	Bio10			
	Mean temperature of coldest quarter (℃)	Bio11			
Water availability	Annual precipitation (mm)	Bio12	2.5 min	1970–2000	WorldClim version 2‐Bioclimatic variables (http://worldclim.com/)
	Precipitation of wettest month (mm)	Bio13			
	Precipitation of driest month (mm)	Bio14			
	Precipitation seasonality	Bio15			
	Precipitation of wettest quarter (mm)	Bio16			
	Precipitation of driest quarter (mm)	Bio17			
	Precipitation of warmest quarter (mm)	Bio18			
	Precipitation of coldest quarter (mm)	Bio19			
Productive energy	Normalized difference vegetation index	NDVI	1 km^2^	2008–2018	Resource and Environment Data Cloud Platform (http://www.resdc.cn/Default.aspx)
Habitat heterogeneity	Altitude (m)	Alt	1 km^2^	2000	National Earth System Science Data Center (http://www.geodata.cn/)
	Altitudinal variation (m)	Valt			
Human disturbance	Population density (people / km^2^)	Pop	1 km^2^	2005–2015	Resource and Environment Data Cloud Platform (http://www.resdc.cn/Default.aspx)
	Gross Domestic Product (yuan / km^2^)	GDP			

**FIGURE 2:**
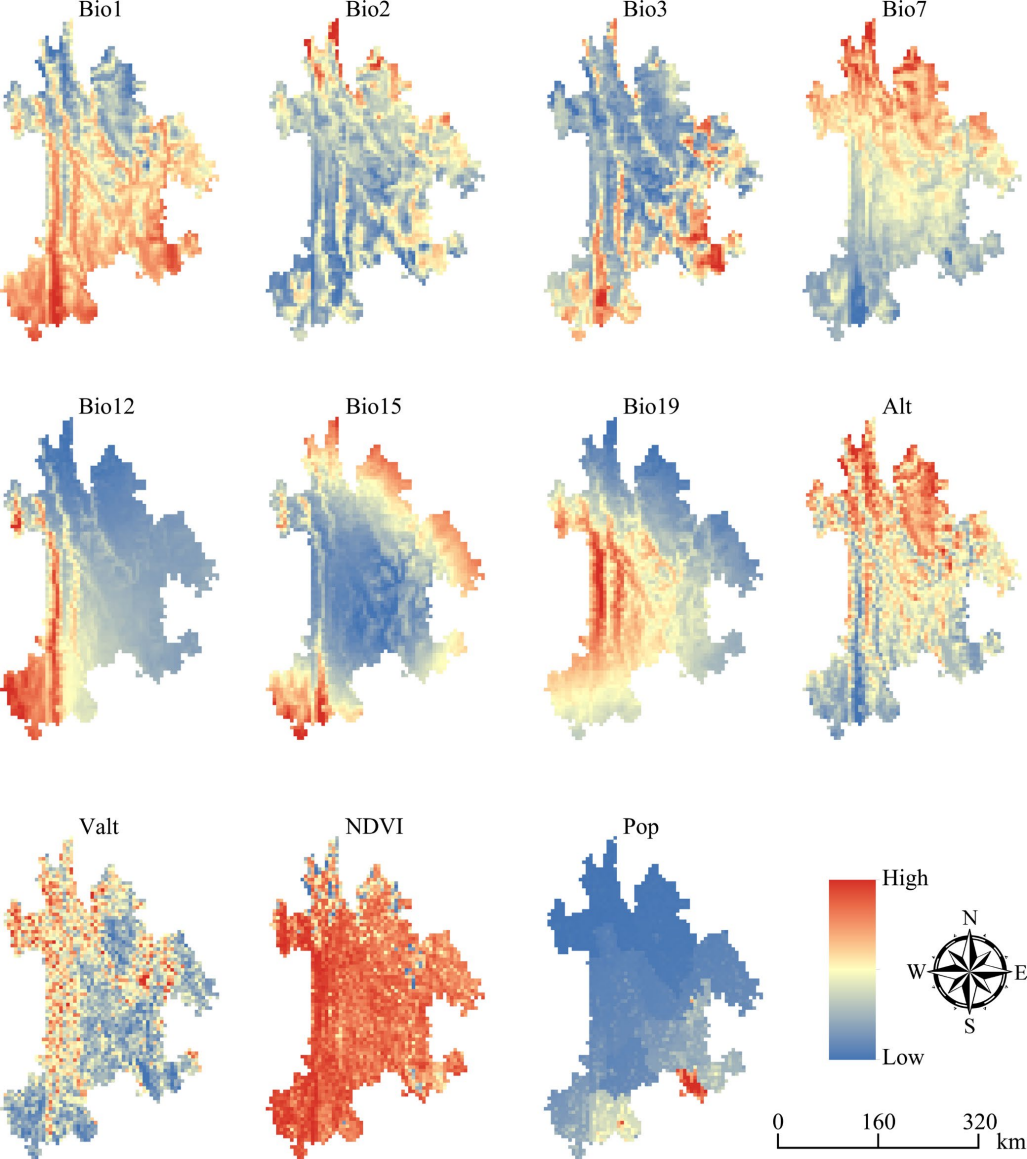
Environmental variables used to model the potential geographical distribution of each species in Northwest Yunnan

In order to avoid multicollinearity of environmental parameters that might result in model over‐fitting, Pearson correlation coefficient (*r*) was calculated between each variable in R 3.5.2 software (https://www.r‐project.org/) (the Pearson correlation coefficient between each variable is in the supporting information). After performed a multicollinearity test, 11 environmental variables (|*r*| < .8) were finally obtained to model the potential geographical distribution of each species (Mukherjee et al., 2020; Zhang et al., 2019) (Figure 2).

### 2.3 Model construction

Two models were constructed in this research: One was for predicting the potential geographical distribution area of species; the other was for analyzing the main environmental factors influencing the potential distribution of species.

#### 2.3.1 Construction of the MaxEnt model

In this study, the latitude and longitude of species distribution sites and the 11 environmental factors in Northwest Yunnan were simultaneously imported into the MaxEnt model (version 3.3.3k) to construct the correlation function between species and the environment. Usually, the prediction results of the MaxEnt model are related to some set parameters, such as the max number of background points (BC), regularization multiplier (RM), and feature combination (FC) (Zhu et al., 2018). MaxEnt is applied to run with the following modeling regulations: (1) Linear features were applied for species with <10 distribution records; (2) quadratic features were utilized for species with 10–14 distribution records; and (3) hinge features were employed for species with >15 distribution records (Zhang et al., 2012, 2017). In this research, the RM value was set to [0.5, 3], the step size was 0.5; the BC value set as [5,000, 15,000], the step size was 5,000. After that, the linear, quadratic, and hinge features were applied to construct the MaxEnt model, respectively (the specific value of each parameter used to construct the MaxEnt model is in the supporting information). In addition, 75% of species distribution locations were randomly selected as training data to build the model, and the remaining 25% of the species distribution locations were used as testing data for model validation (Guan et al., 2018; Zhang et al., 2019). The maximum iterations were set as 500, and the number of replicate runs was set as 10 or 20.

#### 2.3.2 Construction of the GWR model

GWR model is a local regression model, which can profoundly explain the spatial nonstationarity relationship between response variables and explanatory variables by decomposing global parameters into local parameters (Tripathi et al., 2019a). The regression equation can be developed as follows (Han et al., 2016; Tripathi et al., 2019b):

$$y_{i}=\beta_{i0}\left(u_{i},v_{i}\right)+\sum_{k=1}^{p}\beta_{\mathrm{ik}}\left(u_{i},v_{i}\right)x_{\mathrm{ik}}+\varepsilon_{i}$$

where *k* = 1, *p* explanatory variables, *ε_i_* denotes the random error term at position *i*. In addition, (*u_i_*, *v_i_*) represents the geographic coordinate or spatial location of each observation, *β_i_*_0_ (*u_i_*, *v_i_*) is the intercept at position *i*, and *β_ik_* (*u_i_*, *v_i_*) denotes the local regression coefficient at position *i*. When *β*_1_ *_k_* = *β*_2_ *_k_* = … = *β_nk_*, it indicates that the GWR model is transformed into an ordinary linear regression model. In this study, the potential species richness within each grid was used as dependent variables and environmental factors were used as independent variables to investigate the explanation capabilities of different categories of environmental parameters on the potential geographical distribution patterns of species.

According to Tobler's first law (TFL) of geography (Tobler, 1970), the basic principle of the GWR model to calculate the weight is “the closer the distance, the higher the assigned weight; on the contrary, the lower the assigned weight (Fotheringham et al., 2002).” Therefore, the weight can be calculated by a monotonically decreasing function in space distance with [0, 1] as the value range. This type of function is called as the “kernel function” (Lu et al., 2020). The GWR method usually employs a Gaussian model as a weight function, where bandwidth is a function that describes the weight and the distance and is considered as an important control parameter in weight calculation (Gao et al., 2019). The function is expressed as follows (Wang et al., 2020):

$$\omega_{\mathrm{ij}}=\exp\left(‐\frac{d_{\mathrm{ij}}^{2}}{b^{2}}\right).$$

where *ω_ij_* denotes the distance weight of observation location *i* and *j*, *d_ij_* is the Euclidean distance between location *i* and *j*, and b represents the bandwidth. When the distance between location *i* and *j* is larger than b, *ω_ij_* is equal to 0; when the distance between location *i* and *j* is equal to 0, *ω_ij_* is equal to 1.

### 2.4 Model evaluation

#### 2.4.1 Evaluation of the MaxEnt model

Area under the receiver operating curve (AUC) has emerged as the most popular in the MaxEnt literature (Merow et al., 2013; Schroth et al., 2015). AUC is a threshold independent measure of predictive accuracy based only on the ranking of locations (Merow et al., 2013). AUC value was adopted to evaluate the fitting accuracy of the MaxEnt model. The model fitting accuracy can be evaluated as failed if AUC value is between 0.50 and 0.60, poor if AUC value is between 0.60 and 0.70, fair if AUC value is between 0.70 and 0.80, good if AUC value is between 0.80 and 0.90, and excellent if AUC value is between 0.90 and 1.00 (Phillips et al., 2006; Zhang et al., 2019). In addition, the suitability maps were calculated employing the logistic output of the MaxEnt, and the range of habitat suitability index (HSI) value obtained was [0, 1]. According to a large number of previous studies and the expert experience method (i.e., this method of classification has been used in a good deal of studies), HSI value was reclassified into four grades by Natural Breaks in ArcGIS 10.4 software: [0, 0.20] is low, [0.20, 0.40] is medium, [0.40, 0.60] is high, and [0.60, 1.00] is optimal (Ansari & Ghoddousi, 2018; Convertino et al., 2014; Yi et al., 2017; Zhang et al., 2019). In order to conservatively estimate the suitable potential geographical distribution area of species, grids with the HSI value larger than or equal to 0.40 might be considered as the suitable potential distribution area.

#### 2.4.2 Evaluation of the GWR model

In this study, 11 environmental variables were classified as 6 different models (i.e., temperature model, water model, productive model, topographical model, human activity model, and comprehensive model) and investigated which of them was the best predictor on the potential distribution patterns. Furthermore, bandwidth is an important parameter for GWR, which controls the degree of smoothing. In order to choose the best one, the package “spgwr” (Bivand et al., 2020) of R software was used to select bandwidth by adopting Gaussian function and Akaike information criterion (AIC) was employed to confirm the optimal bandwidth. Generally, regression residual is an evaluation value of the fitting goodness of the model, including residual sum of squares (RSS) and residual standard deviation (Sigma), and these two values should be as small as possible. *R* ^2^ denotes the proportion of the variance in the dependent variable that is explained or predicted by linear regression and the independent variable (also known as the predictor variable). In addition, *R* ^2^ and AIC value can also reflect fitting goodness of the model. The higher *R* ^2^, and the lower AIC value, indicating the better fitting effect of the model (Li et al., 2017; Liu et al., 2019). When the difference in the AIC value (∆AIC) of the two models is greater than three, then the model with smaller AIC value reflects a better fitting effect (Han et al., 2016; Xue et al., 2020).

## 3 RESULTS

### 3.1 Species distribution records

On the basis of the selection criteria in section 2.1, 25 rare and endangered plant species were finally identified with high simulation accuracy, belonging to 23 genera and 19 families (Table 2; Table S1). The collected species distribution data were organized to obtain a total of 314 distribution records of species, and further lead to understanding the distribution status of each species in Northwest Yunnan. The result suggested that there were at least four distribution records for each species; as a result, detailed distribution records were shown in Table 2. Among them, *Dipentodon sinicus* and *Psammosilene tunicoides* had more occurrence records, with a total of 59 and 41, respectively. They were mainly distributed in the vicinity of the Gaoligong Mountain National Nature Reserve and several PNRs (i.e., Napahai, Bitahai, Haba Snow Mountain, and Yulong Snow Mountain). However, *Anisodus tanguticus*, *Camellia reticulata,* and *Paris rugosa* owned few distribution records, with four for each. They were scattered in the vicinity of Gaoligong Mountain, Baima Snow Mountain, and Cangshan Erhai NNRs.

**TABLE 2:** The checklist of species used in this study was based on IUCN Red List criteria and the National Key Protected Wild Plants List, specifically: (1) Threat level: IUCN Red List criteria was took as reference, including vulnerable (VU), endangered (EN), and critically endangered (CR); (2) Protection level: 1 represents the National Key Protected Wild Plants List (the first), 2 represents the National Key Protected Wild Plants List (the second), I denotes national first‐level protected species, II denotes national second‐level protected species

Species	Threat level	Protection level	Samples	AUC value
*Actinidia pilosula*	VU	2(II)	9	0.88
*Anisodus acutangulus*	CR	—	14	0.86
*Anisodus tanguticus*	—	1(II)	4	0.93
*Aristolochia delavayi*	EN	—	7	0.80
*Bulleyia yunnanensis*	EN	2(II)	10	0.85
*Camellia reticulata*	VU	2(II)	4	0.97
*Coptis teeta*	CR	2(II)	5	0.88
*Cypripedium guttatum*	EN	2(I)	9	0.90
*Dipentodon sinicus*	—	1(II)	59	0.89
*Diphylax uniformis*	VU	2(II)	6	0.99
*Echinocodon lobophyllus*	CR	2(II)	7	0.81
*Fritillaria delavayi*	VU	—	8	0.91
*Gymnadenia crassinervis*	VU	2(II)	5	0.81
*Magnolia rostrata*	VU	1(II)	5	0.93
*Nouelia insignis*	VU	2(II)	25	0.80
*Ottelia acuminata*	VU	—	7	0.80
*Paris dulongensis*	CR	2(II)	7	0.99
*Paris rugosa*	EN	2(II)	4	0.88
*Psammosilene tunicoides*	EN	1(II)	41	0.83
*Rhodiola atuntsuensis*	EN	2(II)	8	0.86
*Sinopodophyllum hexandrum*	—	2(II)	26	0.85
*Sorolepidium glaciale*	—	1(I)	25	0.80
*Taiwania cryptomerioides*	VU	1(II)	4	0.83
*Terminalia myriocarpa*	VU	1(II)	5	0.99
*Tetracentron sinense*	EN	1(II)	10	0.82

Note

Samples: the distribution records of the 25 rare and endangered plant species as the input of the MaxEnt modeling. AUC value: used to assess the accuracy of the MaxEnt model.

### 3.2 MaxEnt model performance

The average AUC value of each species was between 0.80 and 1.00, indicating that the simulation effect of the model on each species was good or excellent (Table 2). Among them, the minimum average AUC value was 0.80 and the maximum was 0.99 (Table 2), which demonstrated that the constructed MaxEnt model could be used to predict and analyze the potential geographical distribution of 25 rare and endangered species in Northwest Yunnan.

### 3.3 Environmental variable contribution

**FIGURE 3:**
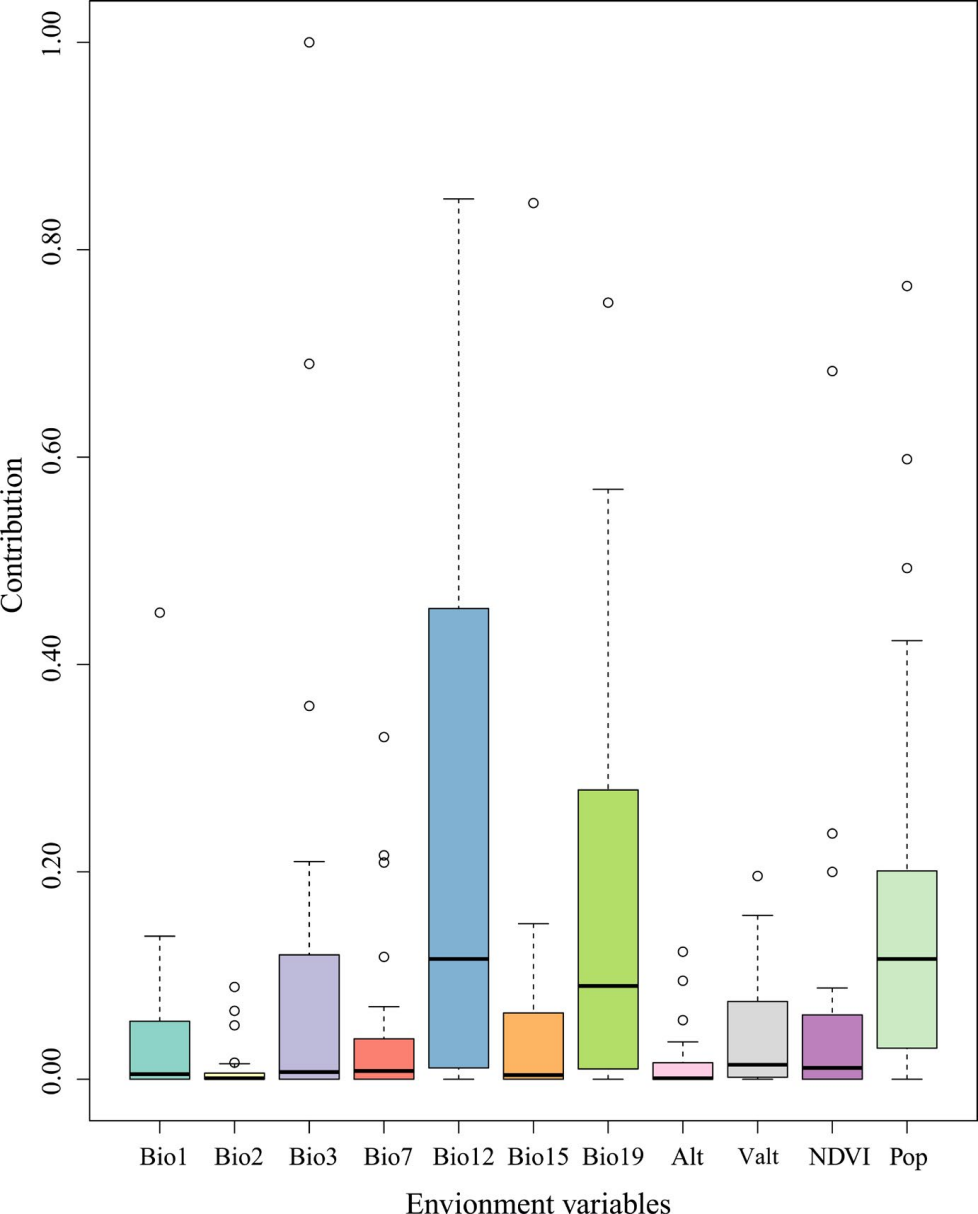
Contribution values of environmental variables to predicting result of the MaxEnt model

The percentage contribution values of environmental variables to the predicted results of the MaxEnt model were defined through heuristics and depended on the specific path that MaxEnt codes used to provide best solutions (Li et al., 2020). In this research, the percentage contribution values were the average contribution values established over cross‐validation and repeated runs for each species (Li et al., 2020). The results showed that among the 11 environmental variables, annual precipitation (Bio12), precipitation of coldest quarter (Bio19), and population density (Pop) made a greater contribution to the species distribution model than other environmental variables (Figure 3). Their average contribution values to the model were 25.92%, 15.86%, and 17.95%, respectively, and the cumulative contribution value accounted for 59.73% of the total contribution value of all environmental factors to the model (Table S2). However, compared with other environmental factors, mean diurnal range (Bio2) and altitude (Alt) made a lower contribution to the species distribution model (Figure 3). Their average contribution values to the model were 1.07% and 1.64%, respectively, and the accumulated contribution value accounted for 2.71% of the total contribution value of all environmental variables to the model (Table S2).

### 3.4 Potential geographical distribution of species

**FIGURE 4:**
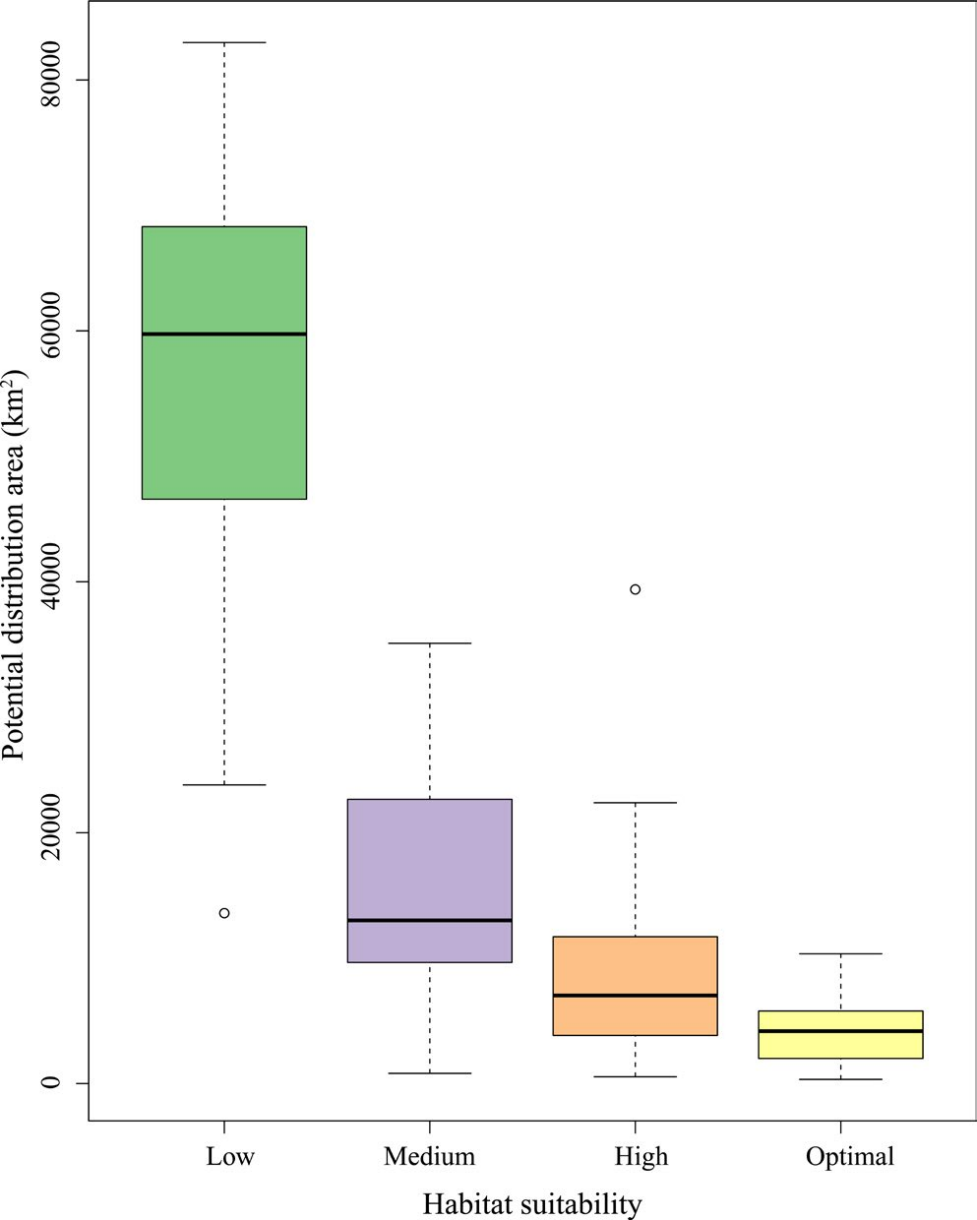
Potential distribution area of habitat with various suitability levels. Different habitat suitability levels show different HSI ranges: HSI is between [0, 0.20] represents low suitability, HSI is between [0.20, 0.40] represents medium suitability, HSI is between [0.40, 0.60] represents high suitability, and HSI is between [0.60, 1.00] represents optimal suitability

**FIGURE 5:**
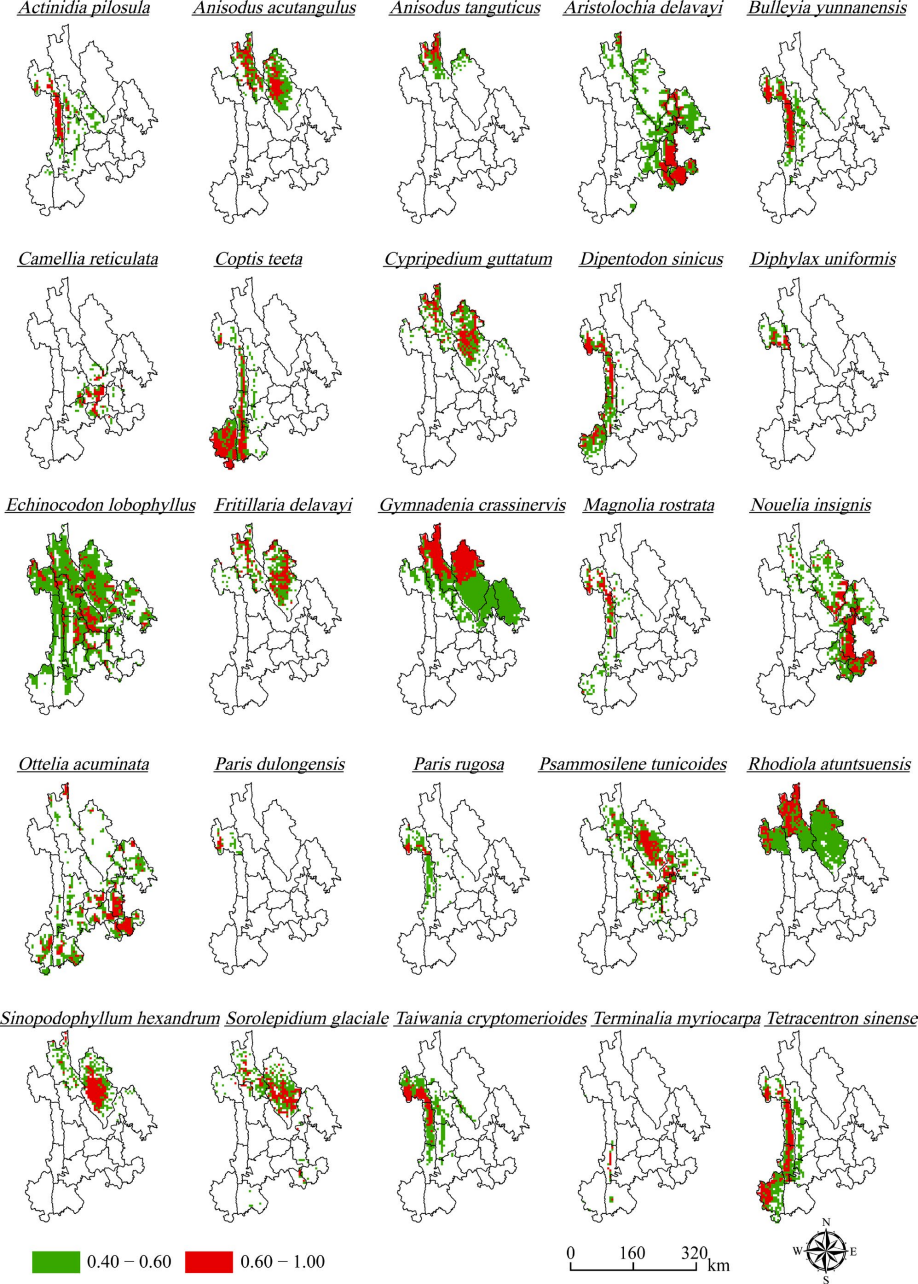
Potential distribution area of each species predicted by the MaxEnt model. The habitat suitability is evaluated as high, if HSI value is between 0.40 and 0.60, and evaluated as optimal if HSI value is between 0.60 and 1.00

HSI is an important or key indicator that affects the survival and development of species. It refers to the potential ability of a habitat to support the survival of a particular species. The results of the study revealed that potential distribution areas of species appear to be decreased with the improvement of habitat suitability (Figure 4), which indicated that the remaining natural habitat suitable for species was becoming less and less. In addition, the research results also indicated that the species with a larger potential distribution areas were *Echinocodon lobophyllus*, *Gymnadenia crassinervis*, *Rhodiola atuntsuensis,* and *Aristolochia delavayi*. Their potential distribution areas occupied 44,963.53 km^2^, 30,830.51 km^2^, 22,481.77 km^2^, and 22,396.28 km^2^, respectively, accounting for 56.36%, 38.64%, 28.18%, and 28.07% of the total area of Northwest Yunnan (Table 3; Figure 5). The species with smaller potential distribution areas were *Diphylax uniformis*, *Paris dulongensis,* and *Terminalia myriocarpa*. Their potential distribution areas occupied 2,137.05 km^2^, 1,054.28 km^2^, and 826.33 km^2^, respectively, occupying only 2.68%, 1.32%, and 1.04% of the total area of Northwest Yunnan (Table 3; Figure 5). Generally, the potential distribution areas of each species were relatively concentrated. The HSI values of the mid‐western, mid‐eastern, and northern regions of the study area were between 0.40 and 1.00, which were deemed the main concentration area of the potential distribution of species. Furthermore, the area of high suitability plaques (i.e., sum area of high suitability level habitats, where 0.40 ≤ HSI < 0.60) was larger than the area of optimal suitability plaques (i.e., sum area of optimal suitability level habitats, where 0.60 ≤ HSI < 1.00) (Table 3; Figure 5).

**TABLE 3:** The area of potential distribution at different suitability levels for each species

Species	Potential distribution area (km^2^)					Proportion of potential distribution area (%)
	Low	Medium	High	Optimal	Suitable	
*Actinidia pilosula*	60,663.73	12,252.42	4,502.05	2,365.00	6,867.05	8.61
*Anisodus acutangulus*	56,019.20	12,223.93	7,294.46	4,245.61	11,540.07	14.46
*Anisodus tanguticus*	69,667.83	5,499.34	3,276.81	1,339.22	4,616.03	5.79
*Aristolochia delavayi*	24,333.88	33,053.04	16,213.09	6,183.20	22,396.28	28.07
*Bulleyia yunnanensis*	49,665.04	21,940.38	4,872.47	3,305.30	8,177.78	10.25
*Camellia reticulata*	70,066.75	5,670.31	2,365.00	1,681.15	4,046.15	5.07
*Coptis teeta*	46,103.29	20,829.11	6,724.58	6,126.21	12,850.79	16.11
*Cypripedium guttatum*	56,275.65	11,283.62	7,778.86	4,445.06	12,223.93	15.32
*Dipentodon sinicus*	60,549.75	9,260.55	6,354.16	3,618.74	9,972.90	12.50
*Diphylax uniformis*	75,737.05	1,909.10	1,396.21	740.84	2,137.05	2.68
*Echinocodon lobophyllus*	12,793.81	22,025.86	37,099.19	7,864.34	44,963.53	56.36
*Fritillaria delavayi*	59,865.89	9,089.59	6,610.61	4,217.11	10,827.72	13.57
*Gymnadenia crassinervis*	22,424.78	26,527.91	21,085.56	9,744.95	30,830.51	38.64
*Magnolia rostrata*	66,391.02	7,892.84	3,618.74	1,880.60	5,499.34	6.89
*Nouelia insignis*	41,629.73	18,606.58	12,936.28	6,610.61	19,546.88	24.50
*Ottelia acuminata*	34,078.82	26,413.94	12,993.26	6,297.17	19,290.44	24.18
*Paris dulongensis*	77,959.58	769.34	712.35	341.93	1,054.28	1.32
*Paris rugosa*	64,339.45	11,568.56	3,276.81	598.37	3,875.18	4.86
*Psammosilene tunicoides*	43,880.76	19,831.82	11,027.18	5,043.44	16,070.62	20.14
*Rhodiola atuntsuensis*	42,199.61	15,101.82	17,039.41	5,442.35	22,481.77	28.18
*Sinopodophyllum hexandrum*	57,728.84	12,936.28	5,328.38	3,789.70	9,118.08	11.43
*Sorolepidium glaciale*	59,096.56	10,371.82	6,382.66	3,932.17	10,314.83	12.93
*Taiwania cryptomerioides*	48,382.81	21,683.93	7,180.49	2,535.97	9,716.45	12.18
*Terminalia myriocarpa*	78,159.04	797.83	512.89	313.43	826.33	1.04
*Tetracentron sinense*	44,935.04	21,342.01	8,348.74	5,157.41	13,506.16	16.93

**FIGURE 6:**
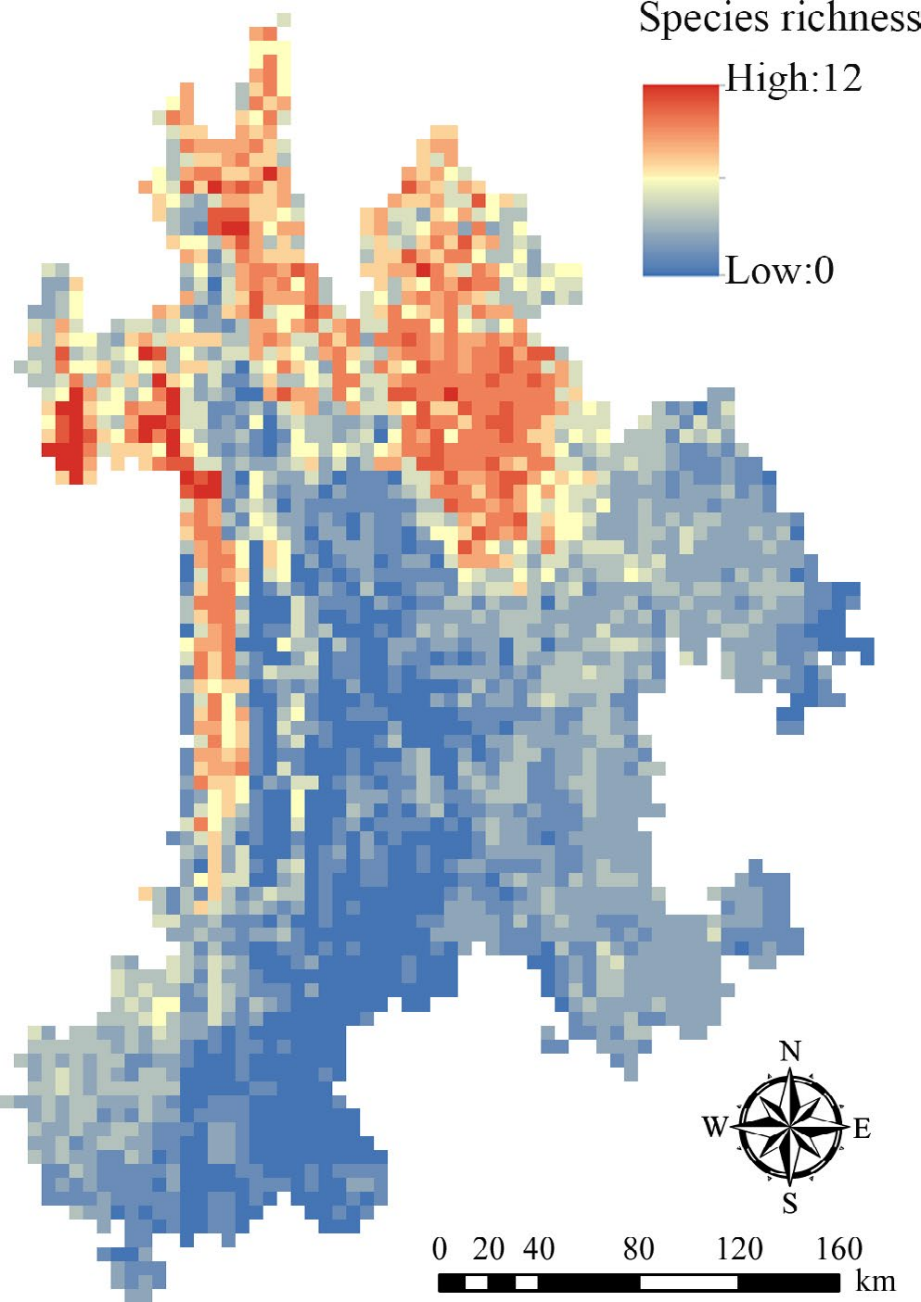
Distribution pattern of potential species richness of 25 rare and endangered plant species in Northwest Yunnan

In addition, the results also showed that on the grid scale with a resolution of 0.05° × 0.05°, the potential species richness of each grid ranged from 0 to 12 (Figure 6). Through a combination of the distribution of the existing national and provincial nature reserves in Northwest Yunnan together with the administrative division of this region (Figure S2; Figure S3), it indicated that part of the grids with the highest potential species richness were located near Baima Snow Mountain and Gaoligong Mountain NNRs, while the other part of the grids with the highest potential species richness were situated in Shangri‐La County (Figure 6).

### 3.5 Environmental explanations for potential distribution of species

According to the category of environmental factors, the package “spgwr” of the software R was employed to construct 6 different types of environmental factor models which might explain the potential distribution of species through the GWR model (Table 4). The research results indicated that the human activity model had a lower interpretation rate than other models, which appeared a goodness‐of‐fit *R* ^2^ and AIC value of 0.83 and 8,850.15, respectively. However, the goodness‐of‐fit *R* ^2^ and AIC value of the water model were 0.88 and 7,703.82, respectively, which gave a higher explanation rate than other models, indicating the water factor largely influenced the potential distribution of species. In addition, the goodness‐of‐fit *R* ^2^ and AIC value of the comprehensive model were 0.92 and 6,785.26, respectively, and the difference in the AIC value (∆AIC) with other models was greater than three, demonstrating that the comprehensive model had a better simulation effect on the potential distribution of species. The results also revealed that multiple environmental variables were not mutually exclusive, and the potential distribution of species was the result of the combined effects of various environmental factors.

**TABLE 4:** Fitting effects of different models on the potential distribution of 25 rare and endangered plant species in Northwest Yunnan. The meaning of the parameters is described in the section of evaluation of the GWR model

Model (0.05° × 0.05°)	Bandwidth (km)	Sigma	RSS	AIC	∆AIC	*R* ^2^
Temperature model	10.37	1.04	2,372.18	7,939.56	1,154.30	.88
Water model	9.45	1.00	2,229.88	7,703.82	918.56	.88
Productive model	6.72	1.15	2,729.22	8,382.65	1,597.39	.86
Topographical model	9.21	1.04	2,376.74	7,900.40	1,115.14	.88
Human activity model	6.64	1.24	3,253.97	8,850.15	2,064.89	.83
Comprehensive model	14.96	0.86	1,528.70	6,785.26	0.00	.92

## 4 DISCUSSION

### 4.1 The potential geographical distribution of species

Habitat is a vital place for survival, reproduction, and population development of species. Its quality might directly influence the distribution, quantity, and survival rate of species (Hall et al., 1997; Zhang et al., 2019). In this research, we utilized the MaxEnt model to predict the potential geographical distribution of 25 rare and endangered plant species in Northwest Yunnan. The results showed that the potential distribution area (i.e., region where HSI ≥ 0.4) of each species was between 826.33 km^2^ and 44,963.53 km^2^, which indicated that these species had obvious differences in their adaptability to environmental factors such as the topography and climate in Northwest Yunnan. The potential distribution area of these target species was mainly concentrated in the mid‐western, mid‐eastern, and northern parts of the study area, which well matched the prediction results of the potential distribution area which studied by Zhuang, Qin, et al. (2018). The prediction results of this study are expected to provide possible new areas for species distribution and field investigation. In addition, the potential distribution area of species overlapped greatly in the mid‐western and northern parts of Northwest Yunnan. We roughly divided them into two parts. One part was close to the Gaoligong Mountain and Baima Snow Mountain NNRs, which were almost consistent with the hotspot distribution of key higher plant species in Northwest Yunnan (Ye, Zhang, et al., 2020), while the other part was located in Shangri‐La County, which might be related to the fact that Shangri‐La is situated in the core area of the Three Parallel Rivers World Natural Heritage Site (Yang et al., 2013), with abundant landscape types, vegetation types, and ecosystem types. In addition, Shangri‐La not only had a very high potential species richness, but also had a very high species endemic rate (Wu, Peng, et al., 2016). Consequently, the results reflected that these regions mentioned above would play a crucial role in biodiversity conservation in the future.

### 4.2 A model used to predict the distribution of species

The model prediction method could help to compensate for the difficulty in field investigation. The vertical peaks and horizontal valleys in Northwest Yunnan greatly limit the accessibility of field surveys. Hence, by combining the model prediction with field investigation, the field survey could be carried out in the order of habitat suitability, with priority given to the field investigation in the optimal suitability distribution area and the potential distribution area that has not been studied. In addition, the completeness, accuracy, and reliability of species geographical distribution data are important and key links of division of key biodiversity areas. In our study, the range of Shangri‐La County was consistent greatly with the potential distribution area of species. This may be related to the sufficient investigation and abundant species distribution data in this area (Wu, Peng, et al., 2016), which further affects the fitting results of the MaxEnt model. Meanwhile, the accuracy of the data and the accuracy of the model prediction are also mutually promoted (Wu et al., 2016; Zhang et al., 2016; Zhuang, Qin, et al., 2018). In this study, we tried our best to collect the species distribution data through multiple approaches, but there are still some species with relatively less distribution data. For example, *Coptis teeta* is also distributed in Myanmar and other countries. Therefore, more species distribution data must be collected to construct a more accurate species distribution model. In addition, the distribution of species is not only determined by topography, climate, and edaphic factors, but also influenced by social and economic structure, land‐use type, human disturbance, and other social factors. In some cases, due to the influence of the local microenvironment, the areas predicted to be of lower suitability levels are actually the distribution areas of species. Therefore, the results obtained in this study are only advisory. A more precise and accurate potential distribution prediction needs to be supported by more comprehensive social and environmental factors and more precise and more reliable species distribution information. In the future, we will evaluate the presence of the target species in areas with high and optimal habitat suitability (a) to look for new geographic locations of the target species and accomplish the population census of the target species and (b) to verify the accuracy of prediction model generated by the MaxEnt in the meanwhile.

### 4.3 Environmental explanations for the potential distribution of species

From an ecological point of view, environmental factors can affect the spatial distribution of species, as well as their habitat suitability (Zhang et al., 2019). In this study, we found that the cumulative contribution rate of the annual precipitation (Bio12) (25.92%), precipitation of coldest quarter (Bio19) (15.86%), and population density (Pop) (17.95%) to the MaxEnt model prediction results reached 59.73%. Furthermore, the contribution rate of the annual precipitation (Bio12) was the highest, which was consistent with the results obtained by Zhuang, Zhang, et al. (2018). In addition, the *R* ^2^ values of the GWR model were all above 0.80, indicating that the model has a reliable goodness of fit for explaining the potential distribution of species. Moreover, the water model (*R* ^2^ = .88, AIC = 7,703.82) showed a higher explanation rate compared with other single models, followed by the temperature model (*R* ^2^ = .88, AIC = 7,939.56) and topographical model (*R* ^2^ = .88, AIC = 7,900.40), which were second only to the water model in their interpretation effect on the potential distribution of species. Accordingly, this result suggested that climate (temperature and water factor) and habitat heterogeneity (topographical factor) could play an important role in the prediction of potential distribution areas of species, which was coincided with the research results of Ștefănescu et al. (2017). Because plant species need a certain temperature and sufficient water in the growing season, hence temperature and water are the main environmental factors that limit the potential geographical distribution of these plant species. In addition, topography is the synthesis of various environmental factors, and temperature and water will change with the terrain gradient and then affect the potential geographical distribution of these plant species. However, compared with all single models, the comprehensive model (*R* ^2^ = .92, AIC = 6,785.26) that combines all environmental factors had a better goodness of fit, which indicated that the potential distribution of species was the result of the combined effects of various environmental factors (Wang et al., 2018). In addition, rare and endangered species are usually restricted to specialized edaphic or topographic or other environmental conditions which occupy a quite fraction of their climatically suitable range. Therefore, on the basis of this study, it is necessary to study the effects of other relevant environmental factors on the potential distribution of species.

### 4.4 Suggestions for the protection of rare and endangered plant species

In recent decades, due to the disturbance of human activities and the impacts of the external natural environment, especially climate change, the population size and distribution area of some rare and endangered species had been declining (Yu et al., 2014). Therefore, understanding the habitat suitability of species and its influencing factors is the basis of protecting rare and endangered plant species (Zhang et al., 2019). Habitat suitability plays an important role in the survival and development of species. Hence, habitat suitability assessment is the first step of effective conservation and scientific management of species and can provide scientific basis for relevant departments to formulate valid conservation strategies. It has become a resultful method to protect rare and endangered species by scientifically predicting the potential distribution areas and habitat suitability levels of species and planning wild nature reserve in the best suitable areas (Xiao et al., 2011; Xu, Cao, Bai, 2015; Xu, Cao, Wu, et al., 2015). In this study, we selected 25 rare and endangered plant species to predict their suitable areas. What matters is that we should pay more attention to the suitable areas of these species, especially the overlapping parts of these species suitable areas, which should be the significant areas for conservation. The results of this study revealed that the habitat suitability of species near Gaoligong Mountain and Baima Snow Mountain NNRs and Shangri‐La Country was relatively high, and these areas were also the core regions for the distribution of rare and endangered species in Northwest Yunnan. For this reason, it is suggested to strengthen the conservation of these areas. In addition, Shangri‐La Country had more habitat suitability distribution areas for species, but there were only three PNRs with a small area, namely, Napahai, Bitahai, and Haba Snow Mountain PNRs (Figure S3). It is recommended that appropriate expansion of the nature reserve should be carried out, with measures combining in situ and ex‐situ conservation to strengthen the protection of species in a state of isolation and/or fragmentation. In addition, the species distribution model is an estimate of the potential distribution of species, and its essence is prediction research. Therefore, the results of the model simulation cannot be used as the only basis for formulating strategies of species conservation, and corresponding field surveys should be carried out according to the simulation prediction results, so as to formulate more scientific and reasonable strategies for species conservation (Renner & Warton, 2013; Wen et al., 2019). Correspondingly, by simulating the potential distribution area of species, it can also provide some basic and scientific evidence for species reintroduction.

## 5 CONCLUSIONS

This study has identified 25 rare and endangered plant species in Northwest Yunnan with high simulation accuracy, belonging to 23 genera and 19 families. The average AUC value of each species was from 0.80 to 1.00, which confirmed that the simulation accuracy of the MaxEnt model on each species was good or excellent. On the whole, the potential distribution area for each species was relatively concentrated, mainly distributed in the central‐western, central‐eastern, and northern regions of Northwest Yunnan. In addition, the potential distribution areas of these species were between 826.33 km^2^ and 44,963.53 km^2^. Additionally, the average contribution values of the annual precipitation (Bio12), precipitation of coldest quarter (Bio19), and population density (Pop) were 25.92%, 15.86%, and 17.95%, respectively. Furthermore, the goodness‐of‐fit *R* ^2^ and AIC value of the water model were 0.88 and 7,703.82, respectively, which demonstrated the water factor largely influenced the potential distribution of these target species. These results would contribute to a more comprehensive understanding of the potential geographical distribution pattern and the distribution of suitable habitats of these target species. This could provide useful information and reasonable reference for us to make recommendations for implementing long‐term conservation, regional management, and reintroduction for these species.

## CONFLICT OF INTEREST

The authors declare that they have no known competing financial interests or personal relationships that could have appeared to influence the work reported in this paper.

## AUTHOR CONTRIBUTIONS

**Pengcheng Ye:** Conceptualization (lead); Data curation (lead); Formal analysis (lead); Investigation (equal); Methodology (equal); Resources (equal); Software (lead); Supervision (equal); Validation (lead); Visualization (lead); Writing‐original draft (lead); Writing‐review & editing (lead). **Guangfu Zhang:** Formal analysis (equal); Methodology (equal); Writing‐review & editing (lead). **Xiao Zhao:** Project administration (equal); Resources (equal). **Hui Chen:** Project administration (equal); Resources (equal). **Qin Si:** Resources (supporting). **Jianyong Wu:** Conceptualization (equal); Data curation (equal); Formal analysis (equal); Funding acquisition (lead); Investigation (supporting); Methodology (supporting); Project administration (lead); Resources (lead); Software (supporting); Supervision (lead); Validation (lead); Visualization (supporting); Writing‐original draft (supporting); Writing‐review & editing (lead).

## Supporting information

Fig S1‐S3

Table S1‐S2

Supplementary Material

## Data Availability

We used open‐access data from WorldClim version 2‐Bioclimatic variables (http://worldclim.com/), Resource and Environment Data Cloud Platform (http://www.resdc.cn/Default.aspx), and National Earth System Science Data Center (http://www.geodata.cn/).
