# Supplementary material for: Potential geographical distribution and environmental explanations of rare and endangered plant species through combined modeling: A case study of Northwest Yunnan, China
Source: Ecol Evol. 2021 Sep 4;11(19):13052–67. doi: 10.1002/ece3.7999 (PMC8495784; doi:10.1002/ece3.7999)
Supplement: Supplementary file 1 — Fig S1‐S3 [file ECE3-11-13052-s002.docx]

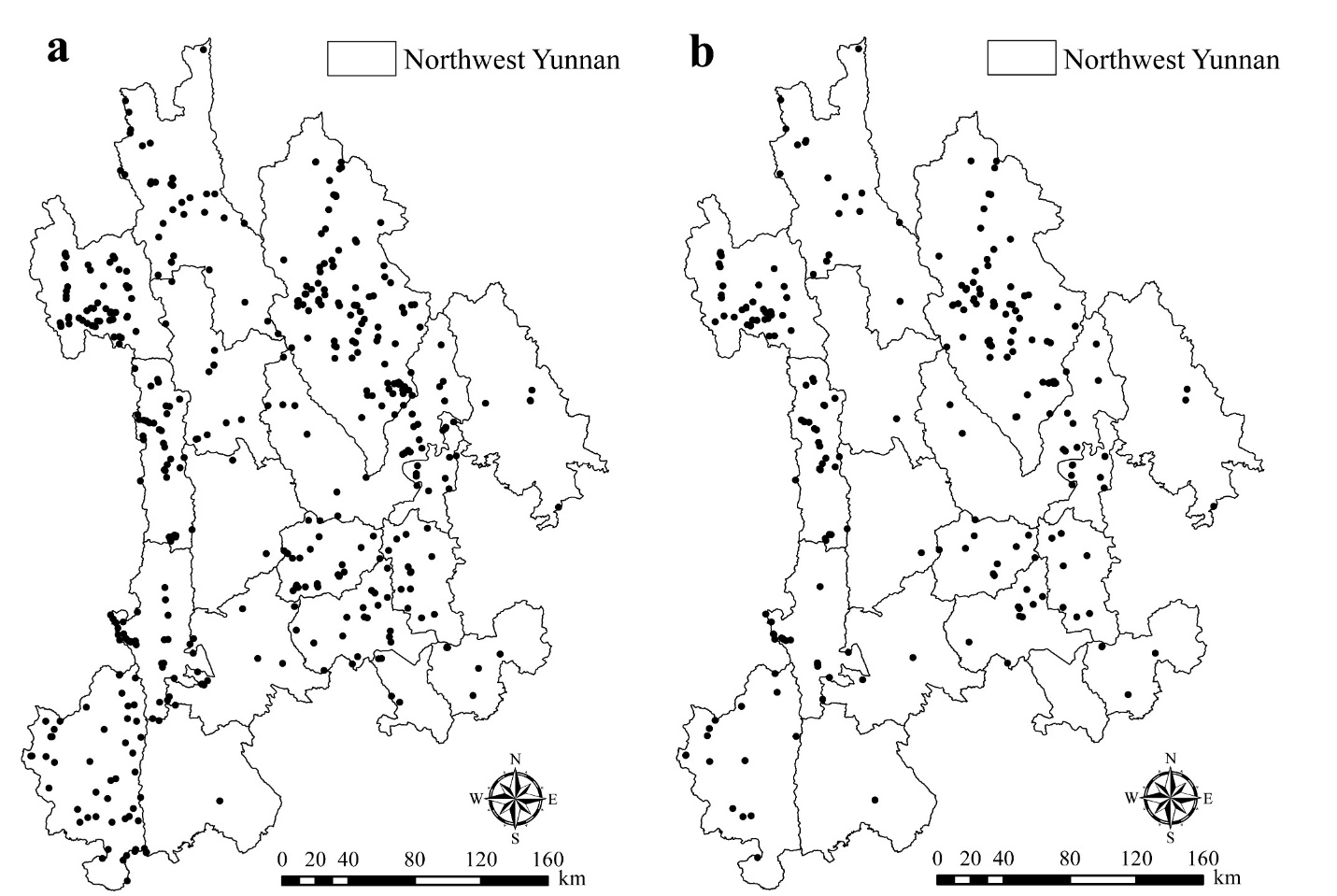


**FIGURE S1** Distribution of the species involved in this study. (a) Before filtering, including 941 distribution records; (b) After filtering or selection, including 314 distribution records.


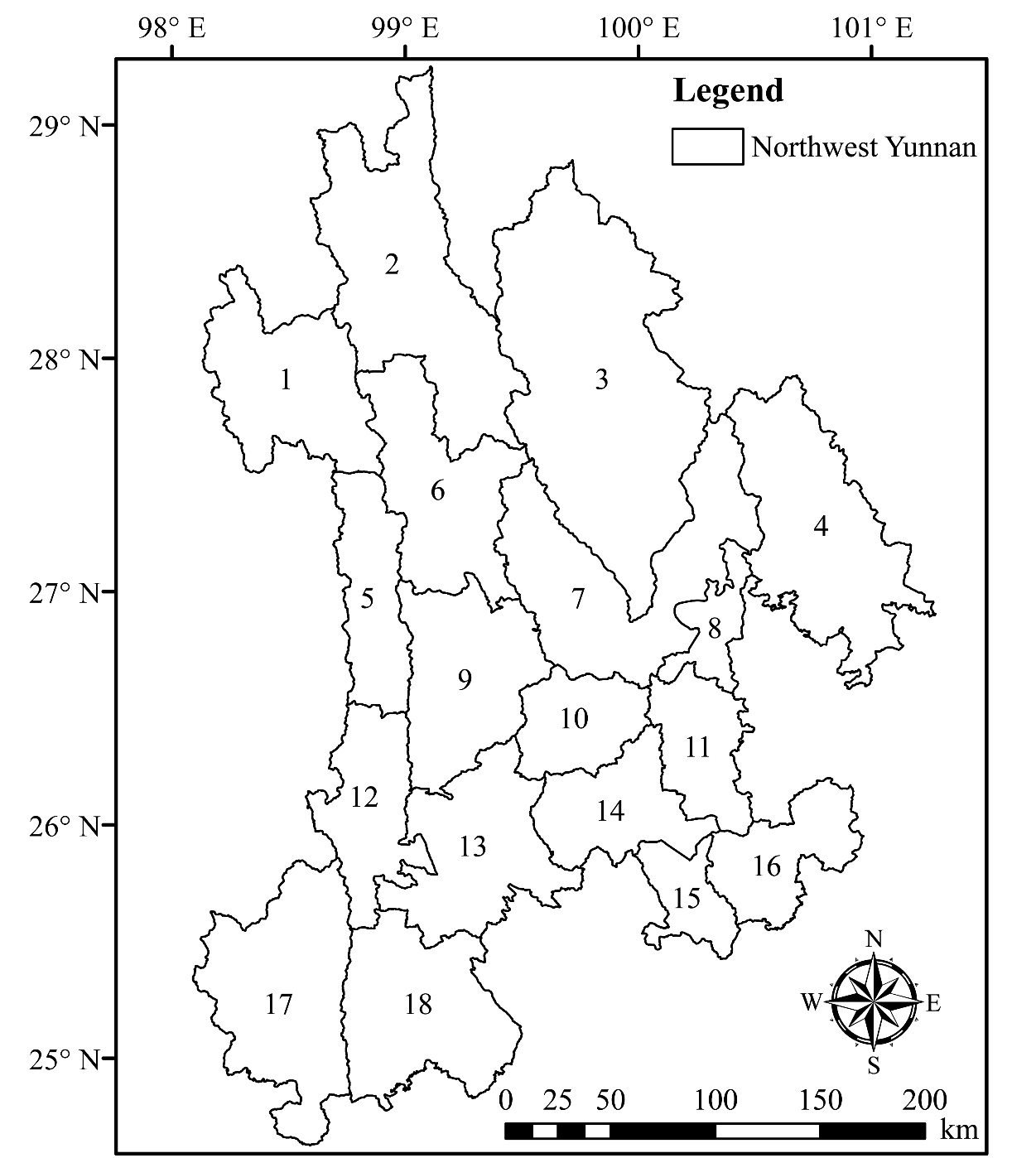


**FIGURE S2** Administrative divisions of 18 counties in Northwest Yunnan. 1: Gongshan; 2: Deqin; 3: Shangri-La; 4: Ninglang; 5: Fugong; 6: Weixi; 7: Yulong; 8: Gucheng; 9: Lanping; 10: Jianchuan; 11: Heqing; 12: Lushui; 13: Yunlong; 14: Eryuan; 15: Dali; 16: Binchuan; 17: Tengchong; 18: Longyang.


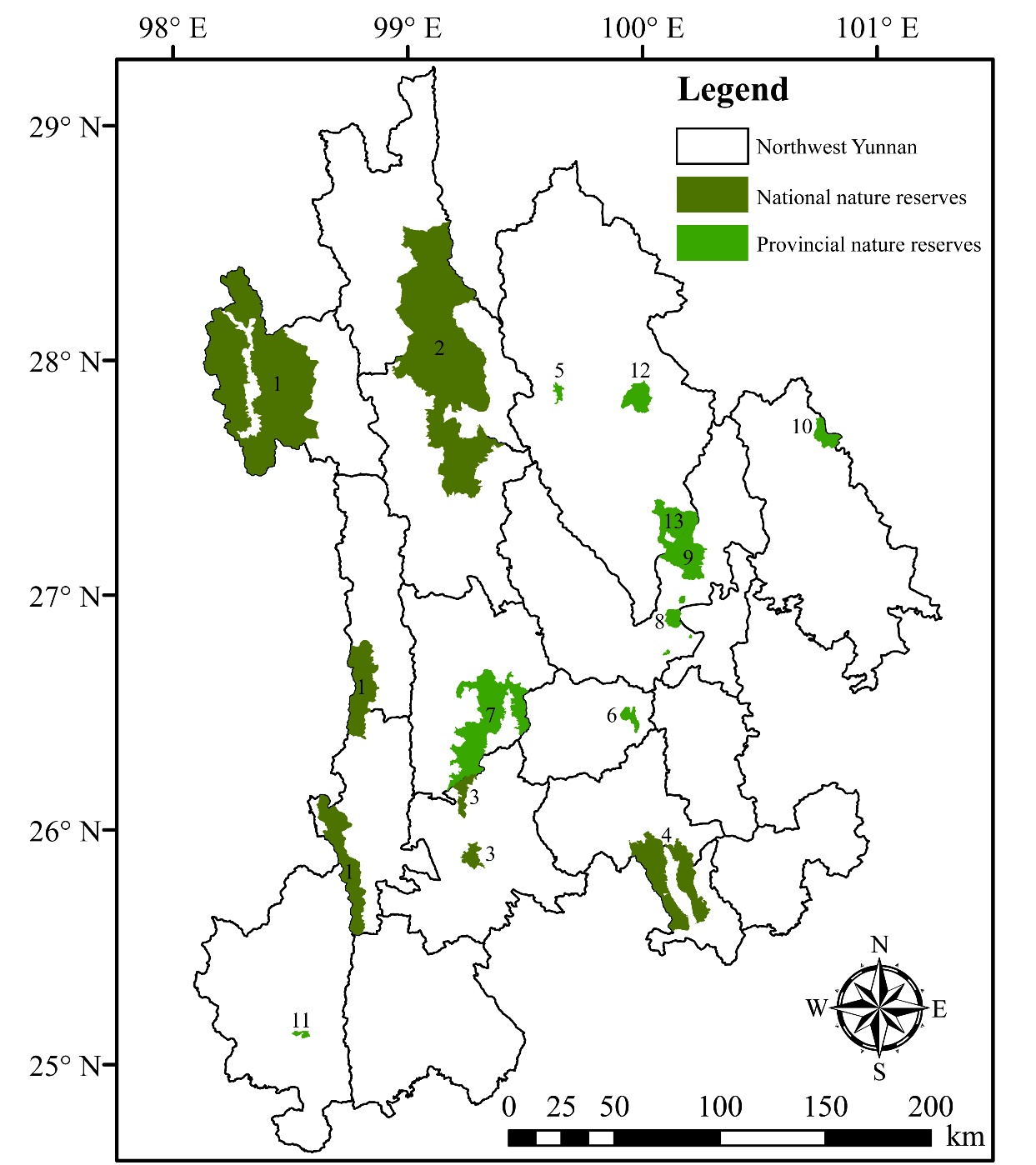


**FIGURE S3** Distribution map of national nature reserves (NNRs) and provincial nature reserves (PNRs) in Northwest Yunnan. 1: Gaoligong Mountain NNR; 2: Baima Snow

Mountain NNR; 3: Yunlong Tianchi NNR; 4: Cangshan Erhai NNR; 5: Napahai PNR; 6: The Sword Lake Wetland PNR; 7: Yunling PNR; 8: Lashihai Plateau Wetland PNR; 9: Yulong Snow Mountain PNR; 10: Ninglang Lugu Lake PNR; 11: Beihai Wetland PNR; 12: Bitahai PNR; 13: Haba Snow Mountain PNR.
