# Supplementary material for: Potential geographical distribution and environmental explanations of rare and endangered plant species through combined modeling: A case study of Northwest Yunnan, China
Source: Ecol Evol. 2021 Sep 4;11(19):13052–67. doi: 10.1002/ece3.7999 (PMC8495784; doi:10.1002/ece3.7999)
Supplement: Supplementary file 2 — Table S1‐S2 [file ECE3-11-13052-s003.docx]

**TABLE S1**Taxonomic information of 25 rare and endangered plant species in Northwest Yunnan. These species belong to 19 families and 23 genera. Among them, there are 1 species of fern and gymnosperm respectively. In addition, there are 23 species of angiosperms, belonging to 17 families and 21 genera. The information for constructing the species checklist mainly derived from: (1) Chinese Virtual Herbarium (http://www.cvh.ac.cn/); (2) *Flora of China* (http://www.iplant.cn/frps). In this checklist, angiosperms are ordered according to Cronquist (1981) classification system.

| Family | Genus | Species |
| --- | --- | --- |
| Dryopteridaceae | *Sorolepidium* | *Sorolepidium glaciale* |
| Taxodiaceae | *Taiwania* | *Taiwania cryptomerioides* |
| Magnoliaceae | *Magnolia* | *Magnolia rostrata* |
| Aristolochiaceae | *Aristolochia* | *Aristolochia delavayi* |
| Ranunculaceae | *Coptis* | *Coptis teeta* |
| Berberidaceae | *Sinopodophyllum* | *Sinopodophyllum hexandrum* |
| Tetracentraceae | *Tetracentron* | *Tetracentron sinense* |
| Caryophyllaceae | *Psammosilene* | *Psammosilene tunicoides* |
| Theaceae | *Camellia* | *Camellia reticulata* |
| Actinidiaceae | *Actinidia* | *Actinidia pilosula* |
| Crassulaceae | *Rhodiola* | *Rhodiola atuntsuensis* |
| Combretaceae | *Terminalia* | *Terminalia myriocarpa* |
| Celastraceae | *Dipentodon* | *Dipentodon sinicus* |
| Solanaceae | *Anisodus* | *Anisodus acutangulus* |
| Solanaceae | *Anisodus* | *Anisodus tanguticus* |
| Campanulaceae | *Echinocodon* | *Echinocodon lobophyllus* |
| Compositae | *Nouelia* | *Nouelia insignis* |
| Hydrocharitaceae | *Ottelia* | *Ottelia acuminata* |
| Liliaceae | *Fritillaria* | *Fritillaria delavayi* |
| Liliaceae | *Paris* | *Paris dulongensis* |
| Liliaceae | *Paris* | *Paris rugosa* |
| Orchidaceae | *Bulleyia* | *Bulleyia yunnanensis* |
| Orchidaceae | *Cypripedium* | *Cypripedium guttatum* |
| Orchidaceae | *Diphylax* | *Diphylax uniformis* |
| Orchidaceae | *Gymnadenia* | *Gymnadenia crassinervis* |

**TABLE S2**Contribution values of environmental variables to the predicted results of the MaxEnt model. Among them, the percentage contribution values were the average contribution values established over cross-validation and repeated runs for each species. Boi1: Annual mean temperature; Bio2: Mean diurnal range; Bio3: Isothermality; Bio7: Temperature annual range; Bio12: Annual precipitation; Bio15: Precipitation seasonality; Bio19: Precipitation of coldest quarter; Alt: Altitude; Valt: Altitudinal variation; NDVI: Normalized difference vegetation index; Pop: Population density.

| Environment variable | Contribution (%) |
| --- | --- |
| Bio1 | 4.45 |
| Bio2 | 1.07 |
| Bio3 | 11.03 |
| Bio7 | 4.67 |
| Bio12 | 25.92 |
| Bio15 | 6.68 |
| Bio19 | 15.86 |
| Alt | 1.64 |
| Valt | 4.36 |
| NDVI | 6.34 |
| Pop | 17.95 |
